# Evolving Management Approaches Toward Personalized Therapy in Acute Myeloid Leukemia: A Narrative Review

**DOI:** 10.3390/jpm16050266

**Published:** 2026-05-15

**Authors:** Pasquale Niscola, Valentina Gianfelici, Marco Giovannini, Carla Mazzone, Maria Ilaria Del Principe

**Affiliations:** 1 Hematology Unit, Medical Area Department, S. Eugenio Hospital (ASL Roma 2), 00144 Rome, Italy; valentina.gianfelici@aslroma2.it (V.G.); giovanninimd@libero.it (M.G.); carla.mazzone@aslroma2.it (C.M.); dlpmlr00@uniroma2.it (M.I.D.P.); 2 Hematology, Department of Biomedicine and Prevention, University Tor Vergata of Rome, 00133 Rome, Italy

**Keywords:** acute myeloid leukemia, age, CPX-351, genomic mutations, fitness, *FLT3* inhibitors, hypomethylating agents, *IDH* inhibitors, measurable residual disease, menin inhibitors, molecular profiling, personalized medicine, prognosis, targeted therapy, venetoclax

## Abstract

After many years of stagnation in the treatment of acute myeloid leukemia (AML), there is currently a rapid move towards personalized medicine. Improvements in molecular diagnostics, risk assessment tools, targeted therapies, overall patient fitness assessments, and quality-of-life assessments have significantly changed how patients are treated. Genetic and molecular analyses, risk and health assessments, and measurable residual disease (MRD) monitoring are now integral to the treatment plan for evaluating patient responses and recurrence. In this regard, lower-intensity treatments are provided to older or unfit individuals. On the other hand, younger patients are usually subjected to curative therapies such as intensive chemotherapy to induce remission. Depending on their fitness and disease risk, they can be considered for hematopoietic cell transplantation, which is done after close observation for MRD. In addition, newer therapeutic drugs and immunotherapy techniques are being applied for patient management. Tremendous strides have been made in improving the efficiency of treatment programs in the relatively new area of personalized AML therapy, with a focus on functionality.

## 1. Introduction

Acute Myeloid Leukemia (AML) is an aggressive blood disorder that mainly affects older adults [1,2]. The disease results from the rapid proliferation of abnormal hematopoietic stem cells (HSCs) driven by genetic mutations that impair normal HSC development within an altered bone marrow (BM) microenvironment (BMM) [3,4]. Therefore, the complex interplay among diverse pathological factors ultimately leads to the development of leukemia stem cells (LSCs) capable of self-renewal and indefinite proliferation [3,4]. Therefore, the concept of AML has changed considerably, shifting from a homogeneous disease to an extraordinarily complex, heterogeneous, and molecularly driven pathological condition [4,5,6]. These advancements have been fueled by developments in genomics and molecular biology and enhanced by new treatments, as well as by changing attitudes toward fitness, quality of life (QoL), individual preferences, and shared treatment decision-making [7,8,9,10]. Additionally, the development of increasingly sensitive methods for detecting measurable residual disease (MRD) in AML has been an essential progress [11]. Indeed, MRD status allows for guided treatment decisions, including whether to escalate therapy with early intensive chemotherapy (IC) or allogeneic cell transplantation (HCT) or to adopt and implement maintenance therapeutic measures, based on relapse risk prediction [11]. Therefore, treatment personalization for patients with AML is a balanced and dynamic clinical synthesis that moves beyond the concept of “one-size-fits-all” IC [1,8,9]. In this view, key approaches include the use of targeted inhibitors for specific mutations, innovative therapeutic combinations, MRD monitoring, and the integration of an early palliative care program from diagnosis onwards to manage physical symptoms and the psychological and spiritual distress of the patient and caregiver [7,8,9,10]. In this context, the objectives of the present narrative literature review on the issue of the current individualized management of AML will be to summarize current knowledge and clinical practice as well as to highlight the areas to be further investigated to glimpse a new horizon of treatment beyond the current effectiveness of modern therapies that are rapidly changing the history and clinical course of this challenging hematologic disease [1,2,8,9,10,11]. Given its biological and clinical peculiarities and specific management, acute promyelocytic leukemia (APL) was not addressed in the present review, as it falls outside our scope; furthermore, high-quality papers reviewing APL and providing the latest advancements in this field are already available in the literature [12].

## 2. Methods

To prepare this review on personalized medicine in cases of AML, PubMed databases were accessed through a search with keywords in areas such as AML, age, CPX-351, fitness, genomic alterations, hypomethylating agents, measurable residual disease, menin inhibitors, molecular analysis, personalized medicine, prognostication, tailored therapies, targeted therapy, and venetoclax. The method adopted in the literature search is iterative and non-systematic, in which selected English-language articles on AML and modern personalized treatment were identified through April 2026. However, the only exception is the study period, which had to be limited to the past two years. The methodological approach adopted here was both dynamic and flexible, guided by field ability that kept evolving with advances in research.

## 3. Key Remarks on the AML Pathobiology

LSCs are found within normal BM niches due to genomic mutations that enable leukemogenesis. This process occurs when HSCs get genetic changes that impair normal blood cell development, leading to the accumulation of immature cells and, if left untreated, potentially rapid disease progression [3,4]. The two-hit theory is one of the mechanisms suggested for acute myeloid leukemia over two decades ago. This theory states that two different genetic mutations must be present for the disease to occur. One mutation influences genes associated with cell growth and division, such as FMS-like tyrosine kinase 3 (*FLT3*) and Rat sarcoma (*RAS*). The second type of genomic alteration affects transcription factors such as Runt-related transcription factor 1 (*RUNX1*), *RUNX1* translocated to 1 (*RUNX1T1*), and the CCAAT/enhancer-binding protein alpha (*CEBPA*), which regulate cell maturation and lead to differentiation arrest [4,13]. Furthermore, epigenetic mutations affecting genes such as DNA Methyltransferase (*DNMT*) 3-alpha, Ten-Eleven Translocation-2 (*TET2*), isocitrate dehydrogenases (*IDH1/2*), and Additional Sex Combs Like 1 (*ASXL1*), as well as spliceosome mutations in Serine/arginine-rich splicing factor 2 (*SRSF2*) and Splicing Factor 3b Subunit 1 (*SF3B1*), are also associated with AML development [3,4]. Notably, all the mutations mentioned are associated with aging and exposure to genotoxic agents, including antineoplastic agents, radiation, and toxins (e.g., benzene), which cause DNA damage and promote clonal hematopoiesis and leukemogenesis [13,14]. From a clinical perspective, the rapid increase in immature and abnormal cells displaces normal HSCs, leading to BM failure, often myeloblastic leucocytosis, and sometimes extramedullary disease, which occur in 1% to 30% of AML patients and are associated with poorer outcomes [15]. Notably, in the pathobiology of AML, alterations in the BMM play a key, complex role in its development [3,4,16,17,18]. These concepts also support the seed-and-soil theory, as AML cells (seed) require a specific BMM (soil) for their development [13]. In this regard, AML cells extensively reorganize the BMM, suppressing normal blood cell production and promoting the development of malignant BM niches in which several pathogenetic factors, including LSCs and BM stromal cells (BMSCs), interact [3,4,16,17]. In addition, it is worth noting that while AML cells adapt the BMM to their requirements, the BMM also exerts pressure on AML cells to develop more resistant, aggressive, and dormant cells, thereby providing a biological basis for leukemic relapse [3,4,11,16]. Moreover, the endosteal (bone-forming) and perivascular (blood vessel-forming) normal niches are also affected [3]. Indeed, AML cells inhibit osteoblast differentiation, thereby forming a pre-osteoblast-rich niche that supports the survival of leukemic blast cells. Again, AML cells stimulate microvascular density (angiogenesis) to provide sufficient nutrients and oxygen to the blast cells [3]. At the same time, their BMMs remain immunosuppressive and hypoxic, showing immunological and metabolic changes that enable AML cells to evade immune responses and resist chemotherapy [16]. Furthermore, hypoxic BMM supports LSCs’ survival and enables them to adapt to stressful conditions by switching between glycolysis and oxidative phosphorylation in nutrient-deprived conditions [19,20]. Indeed, AML cells show intense glycolytic metabolism (the Warburg effect), relying on high glucose uptake and lactate production to support rapid proliferation, even in oxygen-rich environments [20]. This metabolic reprogramming and high lactate production acidify the BMM, thereby causing immunosuppression and chemoresistance and promoting leukemic cell survival and disease progression [3,16,19,21]. The AML cells induce BMMs to express inhibitory checkpoint molecules and produce exosomes that transform BMSCs into supportive cancer cells. It promotes the secretion of cytokines that support cell survival, thereby making the BMM a sanctuary for leukemia development [3,4,16]. Thus, knowledge of BMM biology in leukemia is important for conducting translational studies to formulate a therapeutic approach [3,18]. Currently, leukemia models use the latest technologies, including organ-on-a-chip and hydrogel systems, rather than traditional 2D or suspension culture [18].

## 4. New Classifications and Predictive Models in AML

Advances in the pathogenesis of AML have led to new classification schemes and techniques for assessing risk [11,21,22,23,24]. There is a shift from classical morphology-based classification to a more precise approach using genomic signatures of AML by NGS technologies [6,8]. Thus, AML classification in 2022 has been revamped, considering two new classification systems, namely the WHO 5th Edition (WHO-5) and the ICC 2022 classification systems (Table 1), which differ in blast percentage and terminology [5,6]. The WHO-5 classification system suggested that the BM architecture is irrelevant once the leukemic driver is present in the AML cells [5,6]. However, the ICC criteria have limitations, thereby enabling the classification of transitional forms, namely, the intermediate form of 10–19% blasts in MDS/AML [6]. Specifically, by emphasizing the importance of naming, the ICC2022 offers an opportunity to select the intensity of the AML treatment method [6]. Moreover, the European Leukemia Network (ELN) launched development of an entirely new classification system of prognostic factors, which provides a global consensus on risk stratification based on major molecular markers and their prognostic and therapeutic value [22,23,24]. Additionally, in 2023, the National Comprehensive Cancer Network (NCCN) published guidelines for patients with AML [21]. The main clinical and therapeutic implications, according to the NCCN and ELN2022 guidelines, are summarized in Table 2 [21,22]. It is important to emphasize that the new prognostic scoring system has been confirmed in 1570 patients with AML undergoing cytarabine-based induction therapy [23]. However, the prognostic accuracy of the ELN22 is limited in adults aged 60 and over receiving lower-intensity therapies rather than IC [24]. A multivariable analysis of a large cohort shows that an *IDH2* mutation is an independent positive prognostic indicator, while *KRAS*, *MLL2*, and *TP53* mutations are associated with poorer outcomes. A mutation score was created to classify high-risk patients into two groups: 0–1 point (Beat-AML intermediate) and 1+ points (Beat-AML adverse). The final model merged ELN favorable- and intermediate-risk groups into a new Beat-AML favorable-risk category, with mutation scoring added to the ELN adverse group [24]. This revised risk stratification for older patients with newly diagnosed (ND)-AML provides more precise Beat-AML risk categories that better predict 2-year overall survival (OS) of 48%, 33%, and 11%, respectively [24]. Similarly, a pooled analysis addressing this issue was performed on the phase 3 VIALE-A trial [25,26]. The study showed molecular signatures that distinguish patients treated with the venetoclax-azacitidine (Ven-Aza) regimen by OS, showing the poor predictive power of the ELN 2022 system for differentiating outcomes in this setting. Using bioinformatics, new molecular signatures were found that predicted OS in patients treated with the Ven-Aza regimen [27]. Indeed, mutations in *TP53*, *FLT3*-internal tandem duplication (*ITD*), Neuroblastoma RAS viral oncogene homolog (*NRAS*), *KRAS* classified patients into higher-, intermediate-, and lower- benefit groups (52%, 25%, and 23% of patients, respectively), each linked to a distinct median OS (26. 5 months, 12. 1 months, and 5. 5.5 months) [27]. Although ELN systems are reliable, they encompass only a few genes [28]. New prognostic schemes incorporate extensive clinical and molecular data, including sequencing hundreds of genes, and use machine learning to generate precise predictions [28]. This passage marks the shift from standard prognostic systems at diagnosis to personalized, precise, and dynamic predictions for patients with AML [8,28].

## 5. Fitness Evaluation for the Careful Treatment Eligibility in AML

The combination of molecular profiling and fitness assessment is central to modern AML therapy. While disease biology assesses a treatment’s potential effectiveness, fitness decides its tolerability [9,29]. In this regard, chronological age, as a criterion, does not consistently and reliably reflect the patient’s physiologic reserve, given the variation in functional and cognitive abilities among older populations [29]. However, treatment decisions in AML therapy have long depended on the appropriateness of IC for curative-age patients, typically decided by the patient’s age and Karnofsky Performance Status (KPS) score [9,29,30]. The 2025 ELN and new NCCN guidelines underscore the increasing role of fitness-driven management in AML [9,21]. The standards for judging therapeutic appropriateness have shifted from a basic two-tiered system to a more advanced approach that considers many clinical, biological, and socio-economic variables. The new ELN 2025 guidelines introduce an advanced method of fitness evaluation that helps detect changes in the patient’s health status and offers greater opportunities for individualized treatment [9]. Furthermore, a flexible situational analysis should consider variables such as comorbid conditions, organ function, physical activity, physiological reserve, multimorbidity, frailty, and cognitive status [31,32,33,34,35]. Historically, in the context of AML, the Ferrara criteria have been used to decide patient unfitness for IC [36]. However, according to the Ferrara criteria, patients considered unfit had higher 100-day mortality (42% vs. 5%), as reported in a study of 655 patients [37]. Similarly, an innovative, reengineered, liposomal-encapsulated system to deliver IC, such as CPX-351, which is approved for the treatment of some subtypes of difficult-to-treat AML, was less effective in managing unfit than in fit patients with AML [38]. Furthermore, the Ferrara criteria have proven to be prognostic for newly diagnosed (ND) patients receiving venetoclax combined with hypomethylating agents (HMA), particularly in the “frail” group, where these criteria continue to hold prognostic significance even with low-intensity therapy [39]. These findings underscore the need for a more personalized approach and the importance of thoughtful, multidimensional assessment tools that extend beyond physician judgment [9,29]. Improving the relationship among patients, caregivers, and physicians through PROs and shared decision-making helps align personal goals, such as independence, with treatment plans, especially for aging individuals [7,40,41,42,43,44]. The Comprehensive Geriatric Assessment (CGA) examines overlooked areas of health in older AML patients, including physical, cognitive, psychological, nutritional, and social functioning. Geriatric oncology experts agree that patients aged 65+ with AML should undergo a CGA [33,34,35]. In this regard, Klepin and colleagues found CGA domains predictive of OS and toxicity in patients with AML [33]. Additionally, cognitive assessment must be carried out using the Montreal Cognitive Assessment (MoCA) or the Modified Mini-Mental State Examination (3MS) [43]. Patients’ psychological and emotional status must be assessed, as depression and distress, measured by the Geriatric Depression Scale or the Distress Thermometer, influence resilience and QoL [45]. Therefore, integrating CGA, patient characteristics, and disease profiling is an innovative approach to managing patients with AML [33,34,35,44]. Of note, the SPPB was used to develop a treatment-related score to predict early mortality risk, resulting in 6.8% and 21.9% at 30 and 90 days, respectively, significantly lower than historical data (40%) [44]. Furthermore, the G8 score is an 8-question screening tool that is quick and useful for predicting patient risk and guiding further evaluation. It is useful in differentiating between fit and unfit patients, with median survival of 20.7 and 7.8 months, respectively [45]. Again, nutritional vulnerability is a key issue in AML. Indeed, weight loss and poor nutritional status are risk factors for poor treatment tolerance [46]. However, there are challenges in assessing nutritional status in patients with AML, including the high rate of disease progression, the use of multiple techniques to assess malnutrition, the high prevalence of malnutrition (15–26.5%), and its impact on disease progression [46]. To date, the basic mechanisms of Cancer Cachexia Syndrome (CCS) found in patients with AML are linked to inflammation and cytokine production [46]. Moreover, AML patients on IC face two major challenges, i.e., oral mucositis and neutropenic colitis, further compromising these complications, the nutritional status, making patients vulnerable to nutritional deficiencies, and contributing to the aggravation of CCS [46]. Fitness level can be estimated using methods such as the OPS test, wearables, and continuous monitoring. As for AML, the use of wearables enables continuous estimation of patients’ activity, sleep, and symptom management, especially during the intensive treatment phase. The main goal of using wearables to estimate fitness level is to obtain objective information about patients’ physical condition and activity level. The key periods to monitor include baseline, induction, post-remission, and salvage therapy. Wearables will enable estimation of the degree of inactivity in terms of IC and help recovery in the early stages of IC therapy. Assessment before transplantations and six months post-HCT is aimed at evaluating the level of functional recovery. Moreover, during the follow-up and relapse stages, wearables help detect declines or relapse. Tracking daily step count, sleep duration, and heart rate, as well as higher activity levels, is linked to better recovery. Patient adherence is usually high, and a two-week trial can find non-compliant patients [47,48]. In this regard, the use of accelerometers in wearables has enabled monitoring of patients’ daily activity levels, and OPS was a much better predictor of mortality than clinician- and patient-reported ECOG scores [45].

## 6. The Role of MRD and Its Detection Modalities

Reaching CR is an important and traditional milestone in AML treatment, although novel targeted therapies have changed the significance of traditional endpoints, bringing new treatment goals [49]. Indeed, these new treatments can ensure disease control, including transfusion independence, and support the absence of clinically significant manifestations without conferring a cure [49]. Therefore, long-term OS relies on suppressing residual disease and avoiding relapse. In this regard, extremely sensitive tools enabled quantification of MRD beyond the 5% blast threshold, which is the criterion for morphological CR [11,50]. Currently, three primary platforms are the standard of care: multiparameter flow cytometry (MFC), quantitative polymerase chain reaction (qPCR), and NGS [11,50]. Table 3 shows each approach with specific advantages and technical constraints, while Table 4 illustrates the clinical implications of NGS-based MRD. Additionally, the 2025 ELN-DAVID criteria allow a patient’s response to be classified as best, warning, or high risk for treatment failure (Table 5). Available techniques for detecting MRD enable qualitative classification of MRD responses rather than relying solely on arbitrary thresholds [11,50]. More importantly, the application of MRD monitoring varies with treatment intensity and the clinical milestone being assessed, allowing for personalized therapeutic approaches across various treatment settings, including lower-intensity therapies and patient categories, thereby guiding decisions to go ahead to HCT (Table 6) in suitable patients and to show maintenance treatments [11]. Therefore, moving forward, the discussion of different therapeutic settings for AML will consider the implications of MRD status for treatment decisions, based on the latest developments in understanding MRD in AML [11,51].

## 7. Frontline Treatment Algorithms: Toward a Personalized Approach

**Figure 1:**
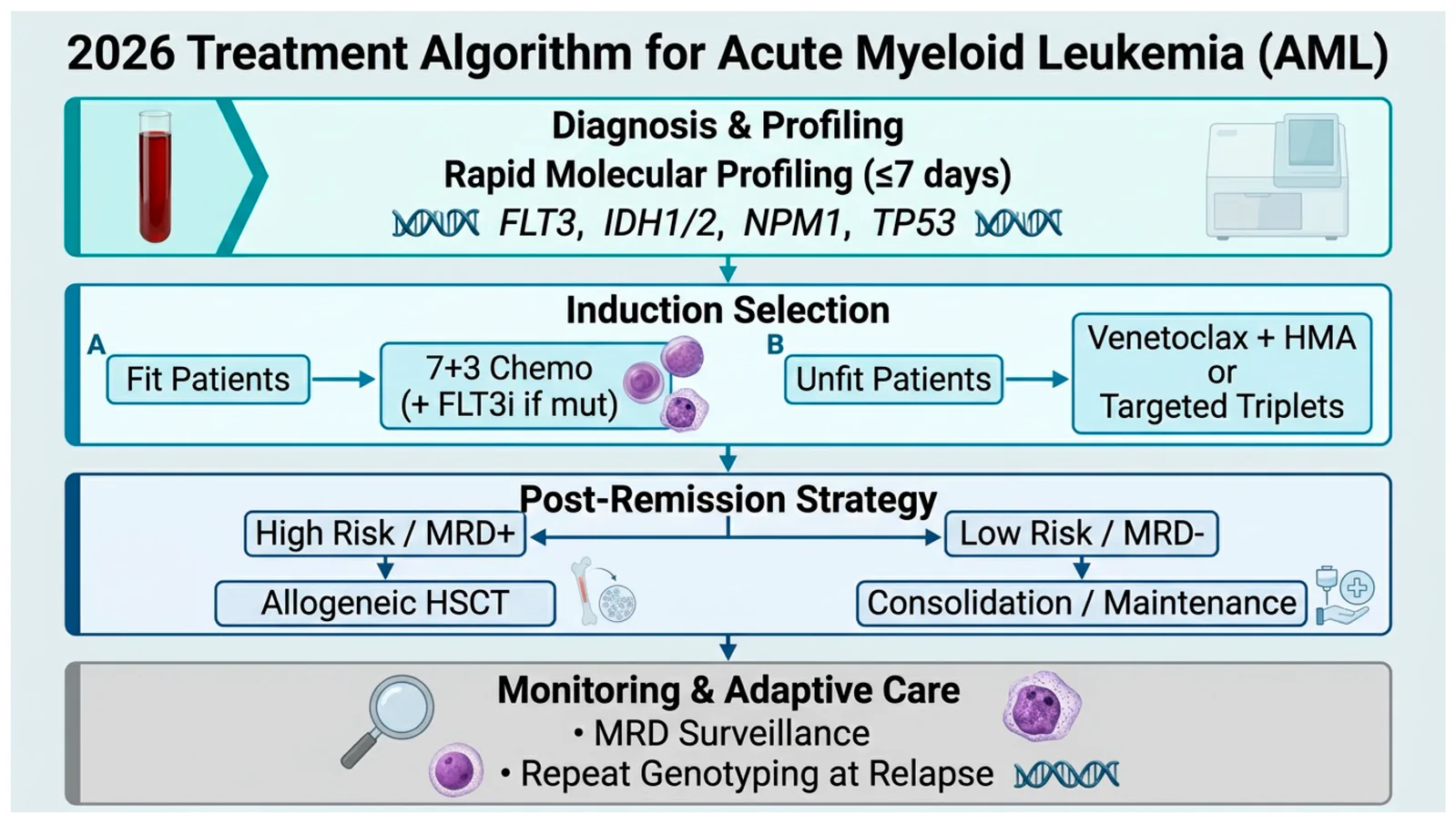
2026 treatment algorithm for AML.

The application of genomic diagnostic techniques in the clinical management of AML has revolutionized initial treatment approaches, particularly for both fit and unfit patients. Today, molecular testing offers individualized treatment regimens, especially for patients harboring mutations in *FLT3*, *IDH1*, *IDH2*, *MLL2*, and *NPM1* genes [52,53,54,55]. Although small-molecule inhibitors were primarily used to treat R/R AML, their advancement toward frontline use is rapidly evolving [1,2,8,55,56]. However, universal molecular profiling at diagnosis remains necessary to identify patients eligible for targeted treatment [8]. In clinical practice, the 7 + 3 induction therapy is the norm for fit patients, with specifically targeted inhibitors, such as midostaurin, quizartinib, added in the presence of *FLT3* mutations, and gemtuzumab ozogamicin when *CBF* AML patients express CD33 [1,8,57,58]. For patients lacking actionable mutations, the treatment approach is based on specific karyotypic aberrations or high-risk criteria [1,8,10]. In this regard, CPX-351 is used as the induction IC in older adults with high-risk diseases, such as therapy-related AML (t-AML) and AML with MDS-related mutations (AML-MR) [59,60,61]. CPX-351 is an innovative IC combination specifically developed for secondary AML by reengineering the traditional 7 + 3 regimen and encapsulating both daunorubicin and cytarabine within a single delivery system [59]. Compared with traditional IC regimens, such as the 7 + 3 regimen, which had unpredictable drug clearance, the liposomal formulation maintains a 5:1 drug-to-cytarabine ratio, thereby enhancing AML cell destruction [59]. Wrapping drugs in liposomes may lower early death rates and toxicities versus standard IC, increasing chances of CR and going ahead to HCT in high-risk AML patients [59,60,61]. Interestingly, these arguments underscore the importance of formulation technologies in personalized medicine. A diverse approach is needed for ND-AML, unfit for IC. More specifically, those that lack actionable mutations usually receive HMAs in combination with venetoclax [52,56]. In patients with IDH1 mutations, azacitidine combined with ivosidenib is the treatment of choice; other potential targeted therapies include gilteritinib, ivosidenib, and enasidenib, depending on the mutation type [2,52,53,54]. Recently, there has been an advance in the form of menin inhibitors as a therapy for patients with *MLL2* rearrangements and *NPM1* mutations [2,56]. All these drugs have received FDA approval or breakthrough therapy designation for R/R AML, in which setting they are also being studied as triplets, and are incorporated into frontline treatments with promising outcomes. Importantly, however, the development of treatments for AML that consist only of oral medications has rapidly developed, particularly among unfit and older patients. Such therapies usually combine HMAs with targeted inhibitors to manage AML effectively while preserving quality of life [55,62,63]. Thus, with new treatment developments, it is imperative to conduct early molecular testing to ensure the proper choice of treatment [52,53]. These concepts inform the current treatment algorithm for AML (Figure 1). In addition, Table 7 summarizes the main findings from AML trials that paved the way for personalized treatment approaches in this setting [25,26,61,62,63,64,65,66,67,68,69,70,71,72].

### 7.1. Updates on Intensive Treatments in Newly Diagnosed AML

The 7 + 3 regimen, which involves 7 days of cytarabine followed by 3 days of an anthracycline, has been the standard treatment for AML for many years, resulting in CR in 60–80% of younger adults and 40–60% of older adults, as reported in a recent analysis assessing treatment time-dependent outcomes over two decades involving 5359 AML patients [73]. Notably, the study results showed that 5-year OS improved over time among different genetic risk groups. For example, the 60-day mortality rate decreased from 13.0% to 4.7%. Moreover, improvements were seen in patients aged <60 years and ≥60 years, with or without HCT. As expected, OS among older adults with AML was poor; however, those aged ≥60 years have a 20% 5-year OS benefit with HCT [73]. However, approximately 40–50% of patients who achieve CR later experience a relapse [73]. The variable effectiveness of IC in clearing the MRD and the LSC population accounts as a key determinant of treatment failure after IC [11]. Indeed, despite CR, leukemia cells at low levels can remain resistant to the first IC, surviving as dominant or secondary clones that may have gotten genetic or epigenetic mutations, thereby making them refractory to further treatment [11,73]. Once more, the biological heterogeneity of AML accounts for its complex, diverse, and polyclonal nature, for which even if initial treatment kills most cells, resistant minor subclones can grow back later. In this regard, patients with *NPM1* mutations usually have better outcomes [73]. In contrast, those with high-risk cytogenetics face a much higher chance of relapse, especially in older individuals with adverse factors, where relapse rates exceed 85% [73]. Additionally, achieving a first CR that lasts less than 12 months is a strong sign of a poor prognosis and higher relapse risk [73]. In this regard, the outlook for patients who relapse after 7 + 3 therapy is generally poor, with an estimated OS of less than 10% at 3 years [73]. Monitoring of MRD helps guide decisions on post-remission therapy, including HCT in first CR of AML with adverse prognostic features [11,74]. Indeed, while salvage IC with similar regimens may be useful, HCT is still the only potentially curative treatment. In this regard, MRD-driven transplant decision-making is an important tool for personalizing the management of patients with AML (Table 5 and Table 6) [11,74]. Another form of IC is CPX-351, used in high-risk/secondary ND-AML, with favorable results compared with the 7 + 3 regimen [61]. Interestingly, a more recent study evaluated whether the OS benefit of CPX-351 over the traditional 7 + 3 regimen is exclusive to specific molecular subgroups of AML using genomic profiling in 184 participants from a randomized phase 3 trial [60]. The trial proved that CPX-351 significantly increased OS in the AML-MR subgroup compared with 7 + 3, with a median OS of 9.7 versus 6.8 months. However, no OS benefit was seen in *TP53*-AML or other AML subgroups. Additionally, in the HCT group, patients receiving CPX-351 had a higher 2-year OS rate of 76%, compared with 27% in the control group. Furthermore, multivariate analysis revealed that CPX-351 and HCT were independent predictors of improved OS in AML-MR. Patients with *TP53*multi showed significantly lower OS than those with *TP53*single (median: 3.8 vs 7.0 months), showing a survival benefit of CPX-351 in AML-MR patients but not in *TP53* patients [60].

### 7.2. IC Incorporating Targeted Agents

Since 2017, targeted therapies combined with conventional IC have started to improve outcomes of patients with AML [74]. Indeed, IC for AML is shifting from the traditional one-size-fits-all approach to a molecularly customized approach that incorporates targeted therapy into the 7 + 3 regimen [1,8,10]. Therefore, the major strategies include improving induction and consolidation therapy by targeting the most common genetic mutations, such as *FLT3*, *IDH1/2*, *NPM1*, and *MLL2*, as well as cytogenetic profiles, thereby reducing relapse rates [1,8,10]. In addition, the anti-CD33 antibody-drug conjugate gemtuzumab ozogamicin, the only antibody-drug conjugate approved to treat, alongside 7 + 3 induction IC, *CBF* AML that expresses CD33 [58]. Notably, *FLT3* inhibitors have transformed the initial treatment approach for AML with mutations (Table 7). In this regard, *FLT3* inhibitors prevent *FLT3* receptor autophosphorylation. Type I inhibitors such as midostaurin, gilteritinib, and crenolanib are effective against cells with *TKD* or *ITD* mutations and are less likely to confer resistance to new *TKD* mutations. Conversely, type II inhibitors such as quizartinib and sorafenib do not target *TKD* mutations, which may develop as a resistance mechanism [75,76,77]. In fact, the combination of midostaurin and IC has become the standard for younger, healthier patients [67]. Furthermore, second-generation inhibitors, such as quizartinib and gilteritinib, are included in frontline therapy due to their increased activity, representing significant advances [75,76,77]. Quizartinib demonstrates high affinity for both *ITD* mutants and the wild-type (WT) *FLT3* protein. In addition, quizartinib has proven its potential as monotherapy for the treatment of R/R *FLT3-ITD*-negative AML. In the phase III randomized QuANTUM-First study, the combination of quizartinib with IC, followed by monotherapy, showed improved OS in patients with *FLT3-ITD*-positive ND-AML compared with placebo plus IC. More recently, the phase II QUIWI trial investigated the safety and efficacy of quizartinib as monotherapy and in combination with IC in *FLT3-ITD*-negative patients with ND-AML, resulting in a significantly longer (20.4 vs. 9.9 months) event-free survival (EFS) compared with the control group. In addition, median OS was unreached and 29.3 months in the quizartinib and control groups, respectively; 3-year OS was 60.8% vs. 45.7%, correspondingly. Most frequent adverse events included fever, rash, diarrhea, and mucositis [75]. Again, gilteritinib has been primarily approved for R/R-AML. Still, it is increasingly studied and incorporated into IC induction and consolidation, as well as into post-remission and post-HCT maintenance, for adult patients with high-risk ND-AML, with favorable results [76,77]. In AML with *IDH1* and *IDH2* mutations, the latter generate the oncometabolite hydroxyglutarate (2-HG), which disrupts epigenetic regulatory pathways [71]. In this regard, ivosidenib (*IDH1* inhibitor) and enasidenib (*IDH2* inhibitor) directly inhibit the mutated enzymes, and their incorporation into induction and consolidation regimens also shows promise, as these agents are expected to yield deeper responses [78,79]. Moreover, recent clinical evidence shows that venetoclax can be incorporated into IC, yielding excellent outcomes in *IDH*-mutated AML [80]. Furthermore, revumenib and ziftomenib, which are part of the menin Inhibitors, are also changing the face of AML therapy, especially for *MLL2*-rearranged and *NPM1*-mutated AML, as results show high overall response rates (ORRs) when the IC regimens are combined [72,81]. Other promising Menin inhibitors, such as bleximinib, BN104 (phase I/II), and enzomenib (DSP-5336), are under active investigation [2,81].

### 7.3. IC Incorporating Venetoclax

Venetoclax-based induction regimens combining venetoclax with IC regimens were evaluated for safety and success in patients with ND-AML [82]. The safety and efficacy of the combination of venetoclax and IC regimens, such as the 7 + 3 regimen with the addition of oral venetoclax at 100 mg on day 4, increasing to 200 mg on day 5, and escalating to 400 mg from days 6–11 (DAV regimen), were evaluated on 33 patients with ND-AML aged 18–35 years [83]. The DAV induction achieved CR in 91% of patients after a single cycle. Moreover, 97% of 30 CR patients were MRD-negative. The notable observation with the DAV regimen was the high 1-year OS and RFS rates of 97% and 72%, respectively [83]. Another study assessed the safety and efficacy of adding venetoclax to the CLAG regimen (cladribine, G-CSF, and cytarabine), with or without idarubicin or mitoxantrone, as salvage therapy in seventy-nine patients with RR-AML [84]. The ORR with the addition of venetoclax to the CLAG regimen was high: 77.4% of patients responded, with 71.0% reaching continuous CR and 48.4% being MRD-negative, compared with 47.4% reaching CR and 18.4% being MRD-negative with the CLAG regimen without venetoclax. At 13 months, the median OS and relapse-free survival (RFS) with the CLAG regimen, including venetoclax, were 22.9 and 15.7 months, respectively, and 18.6 and 10.7 months with the CLAG regimen without venetoclax [84]. Moreover, the efficacy and safety of venetoclax in combination with the CLIA (cladribine, high-dose cytarabine, and idarubicin) regimen were reported [85]. Moreover, the combination of venetoclax with fludarabine, cytarabine, G-CSF, and idarubicin (FLAG-IDA) has shown promising results in patients with ND-AML and R/R-AML [86]. In this case, the FLAG-IDA regimen showed promising results in patients with ND-AML and R/R-AML, with an ORR of 97%, a CCR of 95%, and a 90% rate of MRD-undetectable status. Notably, R/R AML patients in their first salvage and with wild-type *TP53* showed good outcomes, with an ORR of 79%, a CCR of 74% (76% MRD-negative), and a 3-year OS of 51%. The FLAG-IDA regimen, combined with venetoclax, achieved CR induction in patients with ND and R/R AML, including those with *TP53*-positive disease. Therefore, the FLAG-IDA regimen combined with venetoclax offers a new treatment choice, especially for fit patients who can undergo IC and may continue to HCT [86]. Studies are being conducted on CPX-351 in conjunction with targeted drugs such as venetoclax, as well as its potential use among younger individuals [87]. In a phase 1b/2 clinical trial, CPX-351 was combined with venetoclax to treat thirty-three adults aged 18 years or older with R/R-AML, achieving an ORR of 46% and a CR rate of 39%. The OS rate at 2 years was 49%, with 73% of these responders undergoing HCT, indicating that the combination of these two drugs is effective in treating R/R-AML, particularly in the first salvage setting [87]. In addition to venetoclax, other *BCL-2* inhibitors have been developed as targeted therapies to enhance apoptosis [82]. Sonrotoclax, incorporated into the 7 + 3 regimen, is under investigation and has shown favorable results, including high rates of MRD-negative CR [88]. Although rarely diagnosed, accounting for about 0.3–3% of cases, *BCR*: *ABL1*-positive AML, an aggressive form of leukemia that typically occurs de novo, portrays a poor prognosis with standard IC alone. Combining tyrosine kinase inhibitors (TKIs, such as imatinib, dasatinib, or ponatinib) with IC or with HMAs and venetoclax can be particularly effective in this setting, especially in older or frail subjects. At the same time, HCT is often recommended for eligible patients to achieve long-term remission [89].

### 7.4. Non-Intensive Treatment for Newly Diagnosed Patients: From HMAs to Ven-Aza Revolution and Combo Therapies

After decades of stagnation in therapy and a dichotomous approach to AML patients, either aggressive treatment or supportive care, the first substantial breakthrough in this setting was the introduction of HMAs into the management of AML [64,65]. These non-intensive agents have become of critical value for treating AML, especially in older or medically unfit patients who cannot tolerate standard IC. They function by mimicking cytosine and being incorporated into DNA, thereby trapping *DNMT enzymes and resulting in hypomethylation and* reactivation of tumor suppressor genes [64,65]. Among the first-generation HMAs, azacitidine and decitabine are the most used. A phase 3 randomized, open-label trial in 488 patients with AML found that, compared with conventional care, azacitidine extended median OS (10.4 vs. 6.5 months), showing it as a useful treatment option, especially for older and unfit patients [64]. Additionally, clinical trials showed that decitabine achieved a higher ORR in older AML patients, with no significant safety differences compared with standard therapies [65]. The next step was to develop synergistic combinations of HMAs with other agents synergistically effective in AML. In this regard, the combination of azacitidine with a *BCL-2* inhibitor, such as venetoclax, is now regarded as the standard treatment for IC-ineligible patients [25,26]. Notably, another major development in this field is the availability of oral decitabine–cedazuridine (DEC-C), which has a pharmacokinetic and safety profile like that of its intravenous counterpart [62]. Importantly, when combined with venetoclax, DEC-C provides a fully oral, effective, and safe treatment option for older or unfit AML patients, achieving ORRs of 64% in the frontline group and 46% in R/R cases [63]. As a result, DEC-C with venetoclax forms a basis for future combinations with other targeted therapies, fostering hope that AML treatment will soon be fully oral. This strategy aims to improve convenience and speed up adoption as a frontline therapy [8,10,55,63,90].

### 7.5. Venetoclax and HMA: The New Standard for the Unfit Patients with AML

The landmark VIALE-A trial showed that Ven-Aza outperforms azacitidine alone in patients unfit for IC, with a median OS of 14.7 months at a median follow-up of 43.2 months and a higher ORR, thereby making it a more effective option for ND-AML patients unable to undergo IC [25,26]. In fact, this combination therapy demonstrates a higher rate of composite complete remissions (CCR) and EFS than HMA alone, particularly in older patients or those with comorbid conditions [25]. Under such circumstances, achieving an MRD-negative status, defined as <10^−3^ by MFC, still retains predictive significance [11]. Indeed, 41% of participants in the VIALE-A study attained CR and achieved MRD negativity, with an OS of 25.1 months compared with 11.6 months among those with positive MRD [11,25]. At present, ELN-DAVID recommends monitoring in cycles 1, 2, 4, and 7 for patients receiving HMA treatment [11]. Emerging evidence suggests that molecular depth of remission can also be achieved with these regimens, particularly in *NPM1*-mutated cases, raising the possibility that some patients may eventually be able to discontinue therapy [91]. Compared with IC, similar results in terms of CCR, OS, and EFS to those achieved with Ven-Aza have been reported in patients aged 60–75 Years [92]. Notably, preliminary results from the PARADIGM study have indicated a recent breakthrough in this context [93]. The trial randomized 172 untreated AML patients to receive either the 7 + 3 or CPX351 regimens or the Ven-Aza combination, with CCR significantly higher in the latter group (81% vs. 55%) [93]. Moreover, there was no 30- or 60-day mortality in the Ven-Aza group, whereas the IC group experienced 3.5% and 4.7% mortality at 30 and 60 days, respectively. Furthermore, the Ven-Aza group showed a considerable improvement in QoL, physical symptom burden, and depression during the first 2 weeks. Again, patients receiving Ven-Aza did not require intensive care during their first hospitalization, unlike 9.8% of those on IC. Of interest, the inpatient stay was significantly shorter in the Ven-Aza group than in the others during the entire hospitalization period (15 days vs. 36 days) and the first six months (41 days vs. 58 days) [93]. Notably, Ven-Aza’s capacity to achieve deep and CR has also affected the transplant process, being 61% of patients in the venetoclax group undergoing HCT, compared with 40% in the IC group [93]. Additionally, a recent meta-analysis comparing CPX-351 and Ven-Aza therapy found no clear superiority of liposomal IC over Ven-Aza in ORR or OS [94].

### 7.6. Targeted Lower-Intensity Therapy Approaches for IDH-Mutated AML

In patients with *IDH1* mutations who are ineligible for IC, ivosidenib combined with azacitidine (Phase 3 AGILE trial) has shown superior OS (24 vs. 7.9 months) [71]. Moreover, in the same treatment-eligibility setting, patients with *IDH2*-Mutant AML treated with azacitidine or enasidenib yielded favorable results [95]. Therefore, these agents are actively used in monotherapy or in combination with HMAs for R/R and unfit frontline patients [71,95]. Given that *IDH*-mutant AML cells often depend on the *BCL-2* pathway, the Ven-Aza regimen is also considered an especially effective strategy in such a setting [82]. *IDH*-triplet therapy has shown excellent results in 60 patients with *IDH*-mutant ND-AML who are not eligible for IC [96]. In this context, Ven-Aza was combined with *IDH* inhibitors in triplet regimens, resulting in CCR of 92% and 95%, and ORRs of 92% and 95%, respectively. With a median follow-up of 27.4 months, the median OS was not reached. Notably, the 2-year OS and relapse rate were 69% and 24%, respectively. Notably, 32% of patients received HCT, and 51% are still in the study. These encouraging findings confirm the need for prospective trials comparing *IDH*-triplet and *IDH*-doublet regimens [96].

### 7.7. Targeted Lower-Intensity Therapy Approaches for FLT3-Mutated AML

As for the specificity of these mutations, we have two main classes of targeted agents: type I *FLT3* inhibitors (midostaurin, gilteritinib), active against both *ITD* and *TKD* mutations, and type II inhibitors (quizartinib, sorafenib), active only against *ITD* mutations. Lower-intensity therapy for patients with *FLT3*-Mutant Disease is based on HMA, with or without venetoclax, and includes a specific *FLT3* inhibitor [57,97]. Such doublets as azacitidine and gilteritinib were also actively tested and found to be effective in the LACEWING trial, showing increased ORR but no increase in OS due to crossover therapy [98]. However, such a doublet is outdated and has been replaced by newer, more efficient combo regimens (triplet) that use *FLT3* mutation-specific medication in addition to the previously described combination of therapies. The drug with the highest efficacy in this case is gilteritinib. This potent second-generation *FLT3* kinase inhibitor effectively targets both *ITD* and *TKD* mutants, achieving a 90% ORR and high MRD negativity [99]. Another scheme considered for lower-intensity therapy combines HMA, venetoclax, and quizartinib, an *FLT3-ITD*-selective inhibitor and type II inhibitor. This triplet has shown a CCR of 94–100% in patients with *FLT3*-mutant ND-AML and approximately 65–78% in R/R cases, including those previously exposed to *FLT3* inhibitors. Notably, a 1-year OS rate of 72% along with MRD negativity in 70–80% of treatment-naive patients [75].

## 8. Post-Remission Treatment Options

A more thorough comprehension of the molecular mechanisms that underpin AML has improved the capacity to detect very low levels of leukemia-specific abnormalities, such as MRD [11,100]. This knowledge is currently guiding the use of maintenance therapy for patients with AML, including those who are MRD-positive before and after HCT [100]. Therefore, post-remission treatment ranges from consolidation intensity to HCT eligibility and genetics-specific maintenance strategies. Allogeneic HCT continues to play an essential part in achieving long-lasting CR, especially in patients with an unfavorable genetic profile and/or partial MRD clearance and clinical responses [1,11,100,101]. In the absence of an HCT, maintenance or long-term low-intensity treatment, including targeted oral agents or HMAs, will be considered [66,102]. Given this, after consolidation, HMAs can help prolong disease control in AML patients unsuitable for HCT, especially in younger adults [66,102]. The presence of MRD following HCT, irrespective of the absolute value, is considered an immediate call for action [11,101]. Additionally, maintenance therapy is essential for all patients with *FLT3*-mutant AML who cannot undergo an HCT [57,97]. In this regard, maintenance with TKIs such as sorafenib or gilteritinib has been shown to prolong RFS in *FLT3-ITD* AML [97]. In addition, gilteritinib has been shown to confer a significant advantage in patients with MRD during the peri-transplant period [77]. Moreover, the identification of MRD recurrence needs a rapid reduction in immunosuppressive medication doses or the use of donor lymphocyte infusions (DLI) to augment the graft-versus-leukemia response [101]. The strategy involving preemptive DLI based on molecular chimerism or MRD monitoring has proven effective in averting clinical relapse [11,101]. For AML patients in CR who cannot undergo HCT, the QUAZAR AML-001 Trial proved that a partially absorbable oral azacitidine (CC-486) is emerging as a new standard for those aged 55 or older who have achieved remission. This oral azacitidine improved OS (24.7 vs. 14.8 months) and relapse-free survival compared with placebo [67]. Interestingly, oral azacitidine reduced baseline MRD positivity to negativity throughout the course of treatment at a higher rate than placebo (37% vs. 19%). Therefore, oral azacitidine has been approved for maintenance therapy in patients ≥55 years old with first CR or CRi following IC who are ineligible for HCT after the results of the QUAZAR-AML-001 trial, which showed that maintenance therapy with this agent significantly prolonged both RFS and OS, while extending the CR duration without affecting the efficacy of subsequent salvage therapies [66,102]. Options for frail or very elderly patients with actionable mutations include single-agent or targeted oral therapies targeting *FLT3*, *IDH1/2*, and menin inhibitors targeting the *NPM1/MLL2* biology, or use in place of venetoclax/HMA or in combination in triplet regimens [103]. Maintenance therapy after HCT with the help of targeted agents is under active investigation, and the decision is personalized based on genetics and relapse risk [74].

## 9. Updates on Relapsed and Refractory (R/R) AML

Despite advances in frontline and maintenance therapies for AML, the overall outlook remains grim, with more than half of patients progressing to R/R disease [104]. Relapses usually occur within the first two years after treatment and involve clonal evolution, whereby the founder clone and its subsequent subclones facilitate the patient’s acquisition of new mutations that confer drug resistance [104]. In this regard, routine monitoring of clone evolution and relapse genotyping to discover novel actionable clones is important for predicting drug resistance and selecting proper therapy after cure [11,104]. HCT remains the leading potential cure for eligible patients. Recent advances in therapy, such as inhibitors of *BCL-2*, *FLT3*, *IDH1/2*, and menin, improve effectiveness and tolerability, allowing for earlier HCT procedures [104]. In the context of R/R AML, Ven-Aza regimen and disease-specific, individual approaches using markers and targeted agents, such as *FLT3* and *IDH* inhibitors, are effective therapeutic methods [54,55,56,104]. Additionally, triplet combinations, such as azacitidine + venetoclax + gilteritinib, are effective in patients with R/R *FLT3*-mutated AML [70]. Menin inhibitors, which can be used to treat *NPM1*-mutant or *KMT2A*-rearranged AML, including agents such as revumenib, ziftomenib, and bleximenib, have demonstrated significant anti-leukemic effects and represent a notable breakthrough in AML management [103]. In this context, revumenib has been approved by the FDA for the treatment of R/R AML with *MLL2* translocations and *NPM1* mutations [72]. Notably, this agent employs a dual-targeting approach, which is important because it reveals a shared upstream vulnerability between two genetically distinct AML subtypes [72]. This merging of targeted pathways expands the potential use of these inhibitors to about 40% of AML patients, identifying the menin axis as a widely effective therapeutic target, for which specific inhibitors are now being actively studied, including in frontline combinations, such as the Ven-Aza regimen [2,82]. Relapse after transplant can happen and is linked to poor outcomes and requires customized strategies. Managing AML relapse after HCT is especially difficult because there is no universally accepted treatment [105]. Currently, numerous targeted immunotherapies, such as CAR-T cells and bispecific and trispecific antibodies, are also being actively studied in the latter challenging setting of AML relapse [101].

## 10. Immunotherapeutic and Antibody-Based Strategies

Immunotherapies are promising treatment approaches for R/R-AML and for targeting LSCs. Indeed, novel monoclonal antibodies targeting *CD123* (pivekimab sunirine) and C-type lectin-like molecule (*CLL-1*), alongside the established use of GO, are now used to target LSCs with minimal toxicity to normal HSCs [101]. Notably, in this field, ICT01, a new anti-BTN3A antibody, has shown promising results when combined with Ven-Aza in patients aged 75 or older who are considered unsuitable for IC due to comorbidities [106]. Another important breakthrough is the therapeutic evolution of Chimeric Antigen Receptor (CAR) T-cell therapy. However, the development of this treatment modality is challenging in AML, given the heterogeneity of leukemic cells, which may not all express the same antigens, and the possibility that HSCs may express antigens such as *CD33*, *CD123*, and *CLL-1*, which could result in high levels of on-target, off-leukemia toxicity [107]. Notably, during preclinical and early clinical trials, CARs that release messenger proteins to activate other immune cells, such as NK cells, have been developed to address resistance [108]. Recently, researchers have engineered T and NK cells with CARs to precisely target antigens in AML [109]. These therapies are now under preclinical and clinical trials. Although NK cells play an important role in targeting aberrant and cancerous cells, they often exhibit dysfunction in blood cancers. CAR-NK cell therapy uses genetically modified NK cells that specifically target tumor antigens [110].

## 11. Unmet Needs and Mechanisms of Therapeutic Resistance

The success of individualized treatment in AML is still threatened by the development of drug resistance and the process of evolution with time. This challenge is addressed through ongoing efforts to identify new targets, notably in the context of the intractable *TP53*-Mutant AML, which remains a high-risk leukemia subtype with an adverse outcome, characterized by genomic instability, complex karyotypes, and a high rate of resistance to IC and targeted therapies, underscoring the evolutionary resilience of *TP53*-Mutant AML [111]. Other approaches that do not rely on a functional apoptotic system, such as anti-*CD123* antibodies or cellular therapies, are currently being developed and may offer hope [111]. Providing prognostic information in a severe disease like *TP53*-mutant MDS and AML is especially challenging. So, physicians should balance hope with realism by discussing possible treatment options, accepting poor prognoses, encouraging adaptive coping in patients, and explaining the nature of the disease [7,111]. An added critical field in the modern AML management is understanding and overcoming venetoclax resistance (Table 8) [82,112].

## 12. Rise in New Strategies

Other important aspects to further develop to personalize treatment management for patients with AML include predicted advances in pharmacogenetics and therapeutic drug monitoring (TDM) [113,114]. Although the therapeutic benefits in clinical practice have so far been rather limited, further advances in these two important fields of clinical research and therapeutic practice are key to improving AML treatment, given genetic variability, differences in treatment response, and issues such as toxicity and relapse. Pharmacogenetics entails assessing genetic differences in enzymes and drug targets that affect responses to drugs such as cytarabine and anthracyclines, as well as to targeted therapies such as midostaurin, ivosidenib, and venetoclax, a *BCL-2* inhibitor [113]. Differences in genes that regulate drug activation/inactivation, including silent and intronic regions, can influence treatment responses and outcomes, such as myelotoxicity, mucositis, cardiotoxicity, and hepatotoxicity [113]. In addition, TDM supports drug levels within the therapeutic window, avoiding subtherapeutic or toxic concentrations. It is especially important for venetoclax, given its fixed doses and interactions with *CYP3A4* inhibitors, notably in older adults. TDM is now recognized as essential for dose optimization in AML [82,112,114].

## 13. Supportive Care in AML

At all stages of patient treatment, adequate supportive care during both induction and consolidation is important to reduce toxicity [7,115,116,117]. These supportive care measures include, among others, infection control practices, blood transfusion, and management of complications such as hemorrhage and mucositis, which generally lengthen hospital stay [46,115]. Neutropenia, on the other hand, raises the chances of fever. For this patient, G-CSF is given to accelerate neutrophil production and shorten the neutropenic period. In addition, a recently published comprehensive meta-analysis examined the effects of prophylactic G-CSF on AML patients after IC, finding that relapse rates, OS, EFS, and mortality were similar regardless of G-CSF use. Therefore, G-CSF does not affect OS, although the meta-analysis highlighted a higher relapse risk in children and in secondary AML patients who have received this stimulating agent [117].

## 14. Conclusions and Future Direction

Over the last twenty years, the approach to AML treatment has evolved significantly, moving away from non-specific cytotoxic chemotherapy towards precision-targeted therapies and cellular therapies. These advancements in AML management now enable personalized care tailored to each patient’s fitness level, treatment preferences, tolerance, and specific AML features. Finally, the patient’s and caregiver’s QoL should be considered, along with shared decision-making that includes the patient’s goals, the medication itself, and congruence with the patient’s values and social network [6,7]. So, the range of suitability, rather than fit-or-unfit labels, ensures that all patients, regardless of age, can receive a treatment regimen suitable to their physiological capabilities [9,118]. By combining host variables for fitness evaluation with the genetic characterization of AML, the promise of precision medicine in this setting—the concurrent administration of the most effective therapy, together with reduced toxicity burden, ultimately translating into survival benefit and preservation of QoL in an increasingly older and more heterogeneous pool of patients—shall finally be achieved. Additionally, the future of AML treatment will be based on the following four pillars. The first will be to combat functional resistance by developing combination treatments, such as triplets or *MCL1/BCL-xL* inhibitors, to prevent apoptotic escape pathways in venetoclax-based regimens [112,119]. The second will be to combat challenging AML subtypes with novel treatments for ultra-high-risk *TP53*-mutated AML, including innovative multi-target immunotherapies and ultramodern cellular therapies [111]. The third will be to improve monitoring by standardizing MRD detection tools and increasing their sensitivity, thereby enabling treatment escalation or maintenance based on molecular response [11]. The fourth is the wider application of all-oral therapy, which is in ongoing development [8,62,63]. Future progress should also aim to expand TDM and pharmacogenetic research and to confirm new markers through prospective clinical trials [113,114]. Again, artificial intelligence and novel technologies will play an ever more extended role in prognostication and in general across all aspects of AML management, supporting the integration of precision medicine into standard practice in this setting [120]. Therefore, the treatment course in AML involves continuous evaluation of individual clinical features and personal performance abilities, thereby guiding incremental personalization and moving closer to the goal of controlling the disease, which has distinct fitness, social, biological, and treatment-specific signatures in each patient [118].

## Figures and Tables

**Table 1:** Summary of AML Classifications (2022).

Feature	WHO 2022 (5th ed.) [5]	ICC 2022 [6]
Blast Threshold (General)	≥20%	≥20%
Genetically Defined AML	No blast minimum needed (except for BCR: ABL1 and CEBPA).	≥10% blasts needed for most recurrent abnormalities.
The “Gray Zone” (10–19%)	Classified as MDS-IB2	Classified as MDS/AML.
MDS-Related AML	AML, MDS-related (AML-MR); replaces AML-MRC.	Divided into AML with MR-gene mutations and AML with MR-cytogenetics.
Secondary AML	Therapy-related, Germline, and Down Syndrome-related are separate categories.	Prior therapy/MDS history is a Diagnostic Qualifier (added to the genetic diagnosis).
CEBPA Mutations	Includes biallelic (biCEBPA) OR single bZIP mutations.	Specifically requires in-frame bZIP mutations.
TP53 Mutated	Not a standalone entity (classified under AML-MR or by differentiation).	Standalone entity: AML with mutated TP53 (requires ≥20% blasts).

WHO 2022 (5th ed.): the 5th edition of the WHO classification of haematolymphoid tumors; ICC 2022: International Consensus Classification of myeloid neoplasms and acute leukemias; AML: acute myeloid leukemia; BCR: Breakpoint Cluster Region; ABL1: Abelson murine leukemia viral oncogene homolog 1; CEBPA: CCAAT/enhancer-binding protein alpha; bZIP: basic region leucine zipper; MDS: myelodysplastic syndromes; IB2: increased blasts-2; Diagnostic Qualifier: nine specific genes (ASXL1, BCOR, EZH2, RUNX1, SF3B1, SRSF2, STAG2, U2AF1, or ZRSR2) and specific chromosomal changes (complex karyotype), del(5q), −7/−del(7q), del(12p), i(17q), −17/del(17p), del(20q), or idic(X)(q13); AML-MR: MDS-related; MRC: MDS-related changes; TP53: Tumor Protein 53.

**Table 2:** Key Molecular Markers and Risk Classification in AML (ELN 2022 Update) [22].

ELN 2022 Risk Category	Genetic Abnormality	Clinical Relevance/Therapeutic Implication
Favorable	Mutated NPM1 without FLT3-ITD	Good prognosis with IC. Emerging target for Menin inhibitors in R/R.
	CEBPA bZIP in-frame mutation	Good prognosis; often responsive to standard IC
	RUNX1:RUNX1T1 fusion	Often seen in younger patients. It generally responds well to HDC, with early molecular reduction in the fusion transcript associated with improved long-term RFS.
	CBFB: MYH11 fusion	While it generally has a high CRR with standard IC, it has a 30–40% relapse risk. Monitoring for MRD is essential, as the presence of other mutations (e.g., KIT or FLT3) can affect the prognosis.
Intermediate	Mutated NPM1 with FLT3-ITD (any AR) or Wild-type NPM1 with FLT3-ITD	Requires addition of FLT3 inhibitor to IC (Midostaurin/Quizartinib).
	IDH1 or IDH2 mutations	Actionable target for IDH inhibitors (Ivosidenib/Enasidenib); often combined with HMA/VEN.
	MLLT3:MLL2 fusion	A specific subtype of AML is often associated with monocytic differentiation.
	Other abnormalities are neither favorable nor adverse.	Normal karyotype and cytogenetic abnormalities with an intermediate prognosis, with relapse or treatment response risks between those of the favorable and unfavorable groups.
Adverse	Mutated TP53 (VAF > 10%)	Ultra high-risk; high resistance; requires clinical trial enrolment (e.g., novel immunotherapies).
	DEK: NUP214 fusion	A unique, high-risk, and aggressive subtype of leukemia (poor prognosis, IC-resistance), making up about 0.5–2% of all AML cases (younger patients).
	MLL2 rearrangements (other than MLLT3)	AML with MLL2, non-MLLT3, rearrangements are recognized as a highly aggressive, high-risk subtype of AML with a poor prognosis (high rates of chemoresistance and relapse)
	GATA2, MECOM rearrangements	Rare (1–2% of cases), highly aggressive AML often shows severe resistance to standard IC. It commonly presents with monocytic differentiation and monosomy 7, and has a poor prognosis, needing urgent HCT to improve survival.
	BCR: ABL1 fusion	Rare (<1%) AML needing TKIs combined with IC and HCT.
	NUP98 rearrangements	More often seen in children (about 5–7%) but also present in adults (around 4–5%). Poor prognosis and resistance to IC.
	−5, del(5q), −7, −17, 17p	High-risk genetic markers are often included within a complex karyotype and linked to a dismal or poor prognosis.
	AML with MDS-related gene mutations (ASXL1, BCOR, EZH2, RUNX1, SF3B1, SRSF2, STAG2, U2AF1 and ZRSR2).	Poor response to standard 7 + 3; CPX-351 is the preferred IC option.
	Complex karyotype	A complex karyotype with three or more abnormalities is present in 10–12% of AML cases. The most frequent unbalanced abnormalities involve deletions on the 5q, 7q, and/or 17p arms of the chromosomes. This form of aggressive AML is usually linked to a poor prognosis and IC-resistance.

ELN: European Leukemia Net; NPM1: nucleophosmin-1; FLT3: FMS-like tyrosine kinase 3; ITD: Internal Tandem Duplication; IC: intensive chemotherapy; R/R: refractory/relapsed; CEBPA: CCAAT/enhancer-binding protein alpha; bZIP; basic region leucine zipper; RUNX1: runt-related transcription factor 1; RUNX1T1: runt-related transcription factor 1, translocated to 1; HDC: high-dose cytarabine; RFS: relapse-free survival; CBFB: Core Binding Factor Beta; MYH11: Myosin Heavy Chain 11; CRR: complete remission rate; MRD: measurable residual disease; KIT: proto-oncogene receptor tyrosine kinase; IDH: Isocitrate Dehydrogenase; HMA: hypomethylating agents; VEN: venetoclax; MLL2: Lysine Methyltransferase; 2AMLLT3: Myeloid/Lymphoid or Mixed-Lineage Leukemia, Translocated to, 3; TP53: Tumor Protein 53; VAF: Variant Allele Frequency; DEK: name of an oncogene and a nuclear protein; NUP214: Nucleoporin 214 gene; GATA2: GATA-binding protein 2; Mecom; MDS1 and EVI1 Complex Locus; HCT: hematopoietic cell transplantation; BCR: Breakpoint Cluster Region; ABL1: Abelson murine leukemia viral oncogene homolog 1; TKIs: Tyrosine Kinase Inhibitors; NUP98; Nucleoporin 98; q: longer arm; del: deletion; −: loss; p: shorter arm; ASXL1: Additional Sex Combs Like 1; BCOR:BCL6 Corepressor; EZH2; Enhancer of Zeste Homolog 2; SF3B1; Splicing Factor 3b Subunit 1; SRSF2: Serine/Arginine-Rich Splicing Factor 2; STAG2: subunit of the cohesin complex; U2AF1: U2 Small Nuclear RNA Auxiliary Factor 1; ZRSR2: Zinc Finger CCCH-Type, RNA Binding Motif and Serine/Arginine Rich 2.

**Table 3:** Comparison between current methods to detect MRD [11,50].

Modality	Target of Assessment	Detection Sensitivity	Clinical Applicability	Standardization Level
MPFC	Aberrant antigen expression (LAIP/DfN)	10^−3^ to 10^−5^	>90% of patients	Moderate; operator dependent
RT-qPCR	Fusion transcripts, NPM1 mutations	10^−4^ to 10^−6^	40% to 50% of patients	High; well-established
Digital Droplet PCR	Specific mutations, fusions, SNVs	10^−4^ to 10^−5^	Patient-specific targets	Emerging, high precision
NGS	Broad mutational landscape	10^−2^ to 10^−6^	>90% of patients	Improving needs UMIs

MRD: measurable residual disease; MPFC: Multiparameter Flow Cytometry; LAIP/DfN: Leukemia-Associated Immunophenotype/Different from Normal; RT-qPCR: Reverse transcription-quantitative polymerase chain reaction; NPM1: Nucleophosmin 1; PCR: Polymerase Chain Reaction; SNVs: Single Nucleotide Variant; UMIs: Unique Molecular Identifiers; NGS: Next-Generation Sequencing.

**Table 4:** NGS-MRD in AML [11,50].

Mutation Category	Associated Genes	Clinical Relevance in CR	Prognostic Value
Initiating/DTA	DNMT3A, TET2, ASXL1	“Founding” or early events in leukemogenesis; often associated with CHIP/persist in CR.	Low; not predictive of relapse
Leukemia-Specific	NPM1, PML: RARA	Clearance is highly correlated with cure	High; gold standard
Late/Transforming	FLT3, IDH1/2, NRAS, KIT	Reflect residual leukemic burden	High markers of imminent relapse
Adverse/Complex	TP53, RUNX1, MDS-related	Often indicative of resistance	Very High; poor prognosis

NGS: Next Generation Sequencing; MRD: measurable residual disease; CR: complete remission; DTA: a common triad of somatic gene mutations: DNMT3A (DNA methyltransferase 3 alpha), TET2 (Tet Methylcytosine Dioxygenase 2) and ASXL1 (Additional Sex Combs-Like 1), CHIP: clonal hematopoiesis; NPM1: Nucleophosmin 1; PML::RARA: Promyelocytic leukemia/retinoic acid receptor alpha; FLT3:Fms-like tyrosine kinase 3; IDH1/2:Isocitrate Dehydrogenase 1 and 2; NRAS: Neuroblastoma RAS Viral Oncogene Homolog; KIT: CD117; TP53: tumor protein 53; RUNX1:Runt-related transcription factor 1; MDS: myelodysplasia.

**Table 5:** Refined response categorization in the 2025 ELN-DAVID framework [10].

Response Category	Definition and Clinical Significance	Clinical Action/Direction
Optimal	MRD burden is undetectable or below levels associated with a favorable prognosis.	Continuation of planned treatment; low risk of relapse.
Warning	MRD is detectable but at low levels and not clearly associated with imminent failure.	Increased monitoring frequency; review of treatment intensity.
High Risk of Treatment Failure	High or rising MRD levels are indicative of impending morphological relapse.	Consideration of preemptive therapy or expedited transplant.

ELN-DAVID: European LeukemiaNet international working group for MRD ASSESSMENT and VALIDATION in AML; MRD: measurable residual disease.

**Table 6:** MRD-guided decision for HCT. Adapted from [10,51].

Clinical Endpoint	MRD-Negative (Pre-HCT)	MRD-Positive (Pre-HCT)	*p*-Value
Median OS	130.6 months	16.0 months	<0.001
Median DFS	109.6 months	7.1 months	<0.001
1-Year CIR	7.3%	33.7%	<0.0001
5-Year DFS	64%	25%	<0.001

MRD: measurable residual disease; HCT: hematopoietic cell transplantation; OS: overall survival; DFS: disease-free-survival; CIR: cumulative incidence of relapse.

**Table 7:** Pivotal Clinical Trials and Targeted Agents in Frontline AML Management.

Year	Target Pathway	Agent(s)	AML Subtype/Eligibility	Pivotal Trial	Key Outcome/Findings	References
2012	*DNA DNMTs*	Parenteral Decitabine	IC-ineligible patients	DACO-016	Higher ORR in older AML patients compared to standard therapies	[65]
2015	*DNA DNMTs*	Parenteral azacitidine	IC-ineligible patients.	AZA-AML-001	Longer OS (10.4 vs. 6.5 months) vs. conventional care.	[64]
2020	*BCL-2/DNA DNMTs*	Venetoclax + HMA	ND, IC-ineligible adults (Unfit)	VIALE-A	Superior OS (14.7 months) vs. azacitidine alone.	[25]
2021	*DNA DNMTs*	Oral Azacitidine	Patients in CR (HCT-ineligible)	QUAZAR AML-001	Maintenance therapy	[66]
2021	*FLT3*	Midostaurin	ND, FLT3-mutated, IC-eligible adults	CALGB 10603 RATIFY	Superior OS when added to 7 + 3 IC.	[67]
2022	*FLT3*	Gilteritinib	R/R FLT3-mutated AML	Phase 3 ADMIRAL trial	Gilteritinib as the standard treatment for R/R FLT3-mutated AML.	[69]
2022	DNA Intercalation and topoisomerase II Inhibition	CPX-351	Secondary AML, IC-eligible patients.	Phase III Study	Improved 5-year OS (18% vs. 8%) compared to 7 + 3 IC.	[57]
2023	*FLT3*	Quizartinib	Adults (18–75 years) with ND *FLT3-ITD*-AML.	QuANTUM-First	Improved OS by IC-quizartinib (± HCT) followed by up to 3 years of maintenance.	[68]
2024	*BCL-2/DNA DNMTs*	Dec-Ced	ND IC/LIT ineligible patients	ASCERTAIN Trial	DEC-C is comparable to intravenous decitabine.	[62]
2024	*BCL-2/DNA DNMTs*	Venetoclax/Oral Dec-Ced	ND-AML is ineligible for IC.	ASCERTAIN-V	First all-oral treatment for AML (CR: 46.5%; OS: 15.5 months; MRD-negativity: 55.1%.	[63]
2024	*FLT3*	gilteritinib, venetoclax, and azacitidine	ND patients IC-ineligible.	Retrospective study	CR/CRi rate of about 90–96% and MRD negativity > 90%.	[70]
2025	*IDH1*	Ivosidenib + Azacitidine	ND IDH1-mutated, IC-ineligible patients	Phase I/II Data	Targeted therapy for IDH-1 mutant-AML (IC-ineligible).	[71]
2025	Menin-*MLL2/NPM1* Axis	Revumenib	R/R *MLL2* rearranged or *NPM1* mutated AML	AUGMENT-101	FDA approval; high ORR R/R-AML.	[72]

AML: acute myeloid leukemia (AML); FLT3: Fms-like tyrosine kinase 3; ND: newly diagnosed; IC: intensive chemotherapy; OS: overall survival; 7 + 3: IC regimen; BCL-2: B-cell lymphoma 2; DNMTs: methyltransferases; HMA: Hypomethylating agents; DNA: deoxyribonucleic acid; CPX-351: encapsulates a synergistic 5:1 molar ratio of liposomal cytarabine and daunorubicin; IDH1/IDH2: Isocitrate Dehydrogenase 1 and Isocitrate Dehydrogenase 2; MLL2: Lysine methyltransferase 2A; ORR: overall response rate; NPM1: Nucleophosmin 1; CR: complete remission; HCT: hematopoietic cell transplantation; Dec-Ced: Decitabine–Cedazuridine; LIT: lower-intensity therapy.

**Table 8:** Mechanisms of Venetoclax Resistance and Strategies to Overcome Them [82,112].

Mechanism Category	Specific Resistance Factor	Consequence/Clinical Effect	Strategy to Overcome
Genetic Alterations	*TP53*, *FLT3-ITD*, *RAS* pathway mutations (*KRAS*, *PTPN11*)	Drive resistance and disease progression; often associated with high relapse risk.	Triplet combinations (VEN + HMAs + *FLT3*i/*IDH*i); Novel immunotherapies.
Apoptotic Bypass	Upregulation/dependence on *MCL-1* or *BCL-xL*	Compensatory anti-apoptotic function, supporting mitochondrial integrity.	Combined therapy with *MCL-1* or *BCL-xL* specific inhibitors.
Differentiation/Metabolic	Monocytic phenotype; enhanced IFN\gamma signaling; lipid metabolism changes	Leukemia cell survival is independent of *BCL-2* inhibition.	Targeted differentiation agents; exploring novel metabolic inhibitors.
Clonal Selection	Founding clone/subclone survival under VEN pressure	Evolution of a dominant resistant subclone at relapse.	Iterative genetic profiling at relapse; multi-drug regimens targeting multiple subclones simultaneously.

*TP53*: tumor protein p53; *FLT3-ITD*: Fms-like tyrosine kinase 3-internal tandem duplication; *RAS*: Rat Sarcoma; *KRAS*: Kirsten rat sarcoma viral oncogene homolog; *PTPN11*: Protein Tyrosine Phosphatase Non-receptor type 11; VEN: venetoclax; HMA: hypomethylating agents; *FLT3*i: Fms-like tyrosine kinase 3 inhibitors; *IDH*i: Isocitrate Dehydrogenase inhibitors; *MCL-1*: Myeloid Cell Leukemia-1; *BCL-xL*: B-cell lymphoma-extra-large; IFN: interferon; *BCL-2*: B-cell lymphoma 2.

## Data Availability

No new data were created or analyzed in this study.
